# Prognostic factors for falls in Parkinson’s disease: a systematic review

**DOI:** 10.1007/s13760-023-02428-2

**Published:** 2023-11-28

**Authors:** Ane Murueta-Goyena, Oier Muiño, Juan Carlos Gómez-Esteban

**Affiliations:** 1 Department of Neurosciences, Faculty of Medicine and Nursery, University of the Basque Country (UPV/EHU), Barrio Sarriena S/N, 48940 Leioa, Bizkaia Spain; 2 Neurodegenerative Diseases Group, Biocruces Bizkaia Health Research Institute, Barakaldo, Bizkaia Spain; 3 Faculty of Medicine and Nursery, University of the Basque Country (UPV/EHU), Leioa, Bizkaia Spain; 4 Department of Neurology, Cruces University Hospital, Osakidetza, Barakaldo, Bizkaia Spain

**Keywords:** Parkinson’s disease, Falls, Gait, Balance, Prognostic factors, Freezing of gait

## Abstract

**Background:**

Falls represent a critical concern in Parkinson’s disease (PD), contributing to increased morbidity and reduced quality of life.

**Purpose:**

We conducted a systematic review to assess the prognostic factors associated with falls in PD, aiming to provide a comprehensive overview of relevant demographic and clinical parameters, and aid neurologists in identifying subsets of PD patients most susceptible to falls and associated injuries.

**Methods:**

PubMed and Web of Science databases were searched for prospective studies assessing factors associated with falls in ambulatory PD patients across different settings, from inception to August 2023. Data extraction was conducted using CHARMS-PF checklist and risk of bias was assessed with QUIPS tool. PRISMA guidelines were followed.

**Results:**

The initial search yielded 155 references. Thirty-four studies, involving a total of 3454 PD patients, were included in the final analysis. The mean pooled age was 67.6 years, and 45.1% were women. PD patients presented mild motor impairment (UPDRS III score 27.8) with mean pooled disease duration of 5.7 years. Gait and balance disorders and history of prior falls emerged as the most consistent predictors of falls across studies. Disease duration, disease severity, dysautonomic symptoms, freezing of gait, frontal cognitive functions, and PD medication dosages yielded inconsistent findings. Conversely, dyskinesias, age, sex, and depression were unrelated to future falls in PD. Logistic regression models were most commonly employed to identify factors significantly associated with falls in PD. Substantial heterogeneity prevailed in the inclusion of confounding factors.

**Conclusion:**

The evidence suggests that previous history of falls, gait disorders, and poor balance are robust prognostic markers for falls in PD.

**Supplementary Information:**

The online version contains supplementary material available at 10.1007/s13760-023-02428-2.

## Introduction

Falls are involuntary incidents that disrupt balance and result in the body coming into contact with the ground or another solid surface. They pose a significant health problem, particularly in individuals aged 65 and above [1], with an even higher prevalence among those suffering from Parkinson’s disease (PD) [2]. As the population continues to age, falls become an increasingly pressing challenge for public health worldwide [3]. It is estimated that between 10 and 35% of falls in this age group lead to fractures, often requiring hospitalization, with hip fractures being the most common at 10% incidence rate [4, 5].

In patients with PD, the annual incidence rate of falls ranges from 45 to 68%, which is three times higher than in healthy individuals [6, 7]. Approximately 50% of falls result in severe secondary injuries, underscoring the importance of identifying the underlying factors contributing to falls to minimize their occurrence [8]. Developing and optimizing therapeutic strategies to prevent falls requires identifying PD patients at risk for falling. Factors that have been identified as potential risk indicators for falls include previous history of falls, occurrence of freezing of gait (FoG), cognitive decline, compromised postural stability and balance, diminished lower limb strength, and reduced gait velocity [7, 9–13]. However, it is important to note that some of these studies are retrospective in nature, potentially susceptible to recall bias. Therefore, a comprehensive review of the current body of evidence derived from prospective studies is warranted to establish robust prognostic indicators for falls in PD. Thus, the aim of this systematic review was to determine the most significant prognostic factors for falls in ambulatory PD patients identified in prospective studies.

## Methods

### Protocol

We designed the systematic review according to the PICOTS system, and reported the results in accordance with Preferred Reporting Items for Systematic reviews and Meta-Analysis Protocols (PRISMA-P) 2015 Statement [14]. The protocol was registered with PROSPERO (registration number CRD42023437145).

### Ethical considerations

Our study only included anonymized data and no personal information was handled or any procedure applied to human beings, therefore, the ethical approval was not required.

### Data sources and search strategy

The search for systematic review was performed in MEDLINE database (via Pubmed) and ISI Web of Knowledge (see Supplemental File for search strategy). The search included articles from database inception to 15^th^ August 2023, with additional articles identified from reference checking [15]. No language restrictions were applied.

Two independent researchers (A.M. and O.M.) screened all titles and abstracts resulting from the electronic databases using Rayyan software [16]. Whole manuscripts were reviewed for article selection based on eligibility criteria. Discrepancies in article selection were addressed via discussion.

### Study selection and inclusion criteria

Articles were eligible for inclusion if their primary aim was to assess the potential association between a candidate prognostic factor (including demographics, psychometric, biometric or others) and risk of future falls in patients with PD recruited in any setting. Further inclusion criteria were as follows: (1) original articles; (2) longitudinal cohort or case–control studies without limitations of follow-up time; (3) prospective studies. Studies including PD patients with Deep Brain Stimulation, patients who required the use of a wheelchair or whose Hoehn & Yahr (H&Y) stage was 5 were excluded.

### Data extraction

Data from included studies were extracted in a spreadsheet based on CHARMS-PF checklist [17]. We gathered information on the following aspects: (1) Source of data; (2) Sample characteristics (eligibility criteria, demographics and clinical variables); (3) Outcome definition, measurement, and timing; (4) Number, and type of measurement of predictors; (5) Sample size; (6) Missing data in outcome or predictors variables and handling of missing data; (7) Summary of results including the non-adjusted and adjusted estimates of statistical analyses, statistical significance, and the included confounding factors.

### Quality assessment

We used The Quality in Prognosis Studies (QUIPS) tool [18] to appraise the quality of included prognostic studies. The QUIPS tool consists of six domains that assess potential sources of bias conducting prognostic studies: patient selection, study attrition, measurement of prognostic factors, outcome measurement, confounding factors, and statistical analysis and results presentation. The adapted QUIPS tool for the current systematic review is provided in Supplemental Material.

### Data synthesis

We assumed heterogeneity on study design, methodological quality, methods for prognostic factor assessment, duration of follow-up, statistical analyses and data presentation across studies. Therefore, a qualitative synthesis of the available evidence was performed. To assess the certainty in the body of evidence of an outcome, the frequency of finding a significant association was assessed, taking into account both sample size and patient characteristics.

## Results

### Study selection and characteristics

**Fig. 1:**
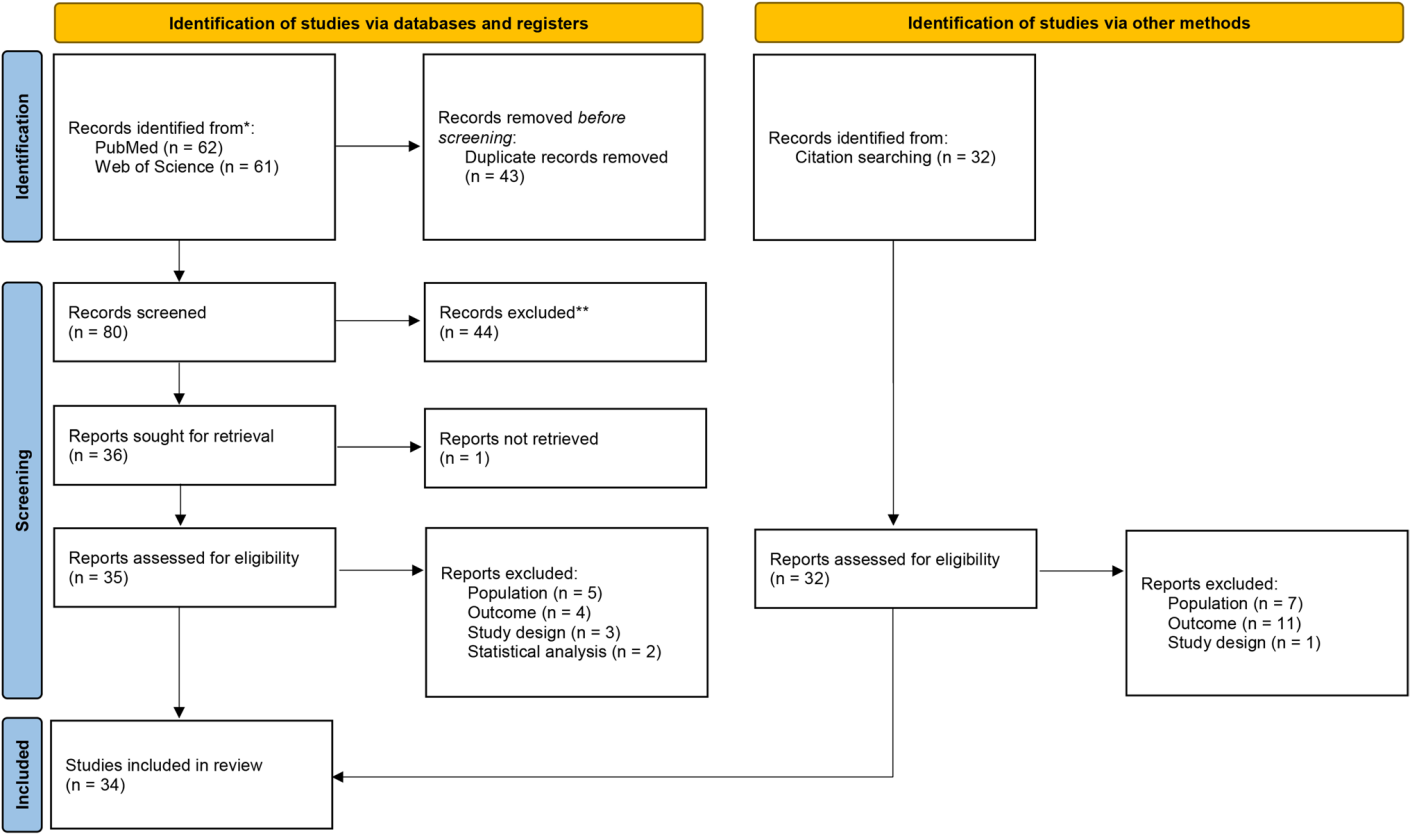
PRISMA flowchart for the study selection

Figure 1 depicts the study selection procedure. The search query retrieved 62 references in PubMed and 61 references in WoS. None of the retrieved papers were in a non-English language. After duplicate removal and screening step, the full text of one article could not be retrieved [19], and thus, 35 references were selected for full text review. Additionally, 32 references were identified by screening reference lists of the selected publications. Table 1 shows the characteristics of the 34 included studies. From these, 31 were cohort studies and 3 were case–control longitudinal studies [20–22]. The follow-up time varied including follow-ups at 3 months [23], 6 months [21, 22, 24–29], 1 year [10, 20, 28, 30–45], 2 years [46], 2 years and a half [47–49], 3 years and a half [50, 51], and 1 study with follow-ups up to 8 years [52].

**Table 1:** Study characteristics

Author	Year	Study design	Country	PD diagnostic criteria	Follow-up (months)	Outcome
Ma et al.	2022	Cohort	China	MDS	6	Falls
Lindholm et al.	2021	Cohort	Sweden	UKPDS-BB	42	Falls, near falls
Van Schooten et al.	2021	Cohort	Australia	198 NA	6–12	Falls
				107 UKPDS-BB		
Lindholm et al.	2020	Cohort	Sweden	UKPDS-BB	42	Falls, near falls
Venhovens et al.	2020	Cohort	The Netherlands	UKPDS-BB	12	Falls
Geerse et al.	2019	Case–control	The Netherlands	UKPDS-BB	6	Falls
Romagnolo et al.	2019	Cohort	Italy	UKPDS-BB	12	Falls
Beretta et al.	2018	Cohort	Brazil	UKPDS-BB	12	Falls
Almeida et al.	2016	Cohort	Brazil	UKPDS-BB	12	Falls, recurrent falls
Cole et al.	2016	Cohort	Australia	UKPDS-BB	12	Recurrent falls
Custodio et al.	2016	Cohort	Peru	UKPDS-BB	12	Falls
Gazibara et al.	2016	Cohort	Serbia	UKPDS-BB	12	Falls
Heinzel et al.	2016	Cohort	Germany	UKPDS-BB	30	Falls
Lord et al.	2016	Cohort	United Kingdom	UKPDS-BB	30	First fall
Sakushima et al.	2016	Cohort	Japan	UKPDS-BB	6	Falls
Schlenstedt et al.	2016	Cohort	Germany	NA	6	Falls
Almeida et al.	2015	Cohort	Brazil	UKPDS-BB	12	Falls, recurrent falls
Duncan et al.	2015	Cohort	USA	UKPDS-BB	12	Falls
Gazibara et al.	2015	Cohort	Serbia	UKPDS-BB	12	Falls
Hoskovcová et al.	2015	Case–control	Prague	UKPDS-BB	6	Falls
Kataoka and Ueno	2015	Cohort	Japan	UKPDS-BB	30	Falls
Hiort et al.	2014	Cohort	Norway	Clinical information in 1^st^ visit, disease development, levodopa response	96	Falls
Kataoka et al.	2014	Cohort	Japan	Calne 1992 and Gelb 1999^a,b^	24	Falls
Mak et al.	2014	Cohort	China	UKPDS-BB	12	Recurrent falls
Duncan et al.	2013	Cohort	USA	Racette et al.. 1999^c^	12	Recurrent falls
Kim et al.	2013	Cohort	South Korea	UKPDS-BB	12	Falls
Mak et al.	2013	Cohort	China	UKPDS-BB	6	Recurrent falls
Paul et al.	2013	Cohort	Australia	NA	6	Falls
Camicioli et al.	2010	Case–control	Canada	UKPDS-BB	12	Falls
Allcock et al.	2009	Cohort	UK	UKPDS-BB	12	Falls
Latt et al.	2009	Cohort	Australia	UKPDS-BB	12	Falls
Mak et al.	2009	Cohort	China	UKPDS-BB	12	Recurrent falls
Wood et al.	2002	Cohort	United Kingdom	UKPDS-BB	12	Falls
Gray et al.	2000	Cohort	Canada	NA	3	Falls

*MDS* Movement Disorder Society, *NA* not available, *UKPDS-BB* United Kingdom Parkinson’s Disease Society Brain Bank

^a^Calne DB, Snow BJ, Lee C (1992) Criteria for diagnosing Parkinson’s disease. Ann Neurol 32:S125–S127.10

^b^Gelb DJ, Oliver E, Gilman S (1999) Diagnostic criteria for Parkinson disease. Arch Neurol 56:33–39

^c^Racette BA, Rundle M, Parsian A, Perlmutter JS (1999) Evaluation of a screening questionnaire for genetic studies of Parkinson’s disease. Am J Med Genet 88:539–543

### Patient characteristics

The total number of included PD patients with follow-ups was 3454. Demographic and clinical characteristics of patients are reported in Table 2. The smallest sample size consisted of 26 patients [46] and the largest one of 305 PD patients [28] who pooled the data from two cohorts [10, 25]. The pooled mean age was 67.6 years old and 45.1% were female PD patients. Disease duration ranged from 0 to 10.2 years (pooled mean 5.7 years), although 8 out of 34 (22.8%) studies did not report this value [10, 20, 21, 23, 28, 33, 37, 45]. Regarding disease stage, Hoenh & Yahr (H&Y) was available in 25 (71.4%) studies, the median ranging from 2 to 3. The pooled mean Unified Parkinson’s Disease Rating Scale (UPDRS), part III score (available in 85.3% of studies) was 27.8. Global cognitive scores were available in 21 out of 34 studies (61.7%), most of them using Mini–Mental State Examination (MMSE), although 2 studies assessed cognition with Montreal Cognitive Assessment (MoCA) [22, 42]. The average cognitive score was normal in all studies for PD patients.

**Table 2:** Clinical characteristics of PD patients

Author	Year	Sample size	Age (years)	Sex (%F)	Disease duration (years)	UPDRS III	HY	Cognition
Ma et al.	2022	51	65.7	35.3	8.0 (4.4)	33.6	2.4	26.1 (3.4)
Lindholm et al.	2021	58	68	55	4.0 (3.9)	12	2	28 (26–29)
Van Schooten et al.	2021	305	68.2	53.1	–	–	–	29.0 (1.6)
Lindholm et al.	2020	73	65	55.1	3.2 (3.7)	10	2	28 (27–29)
Venhovens et al.	2020	30	70	13.3	5	–	2.5	–
Geerse et al.	2019	30	63.1	40	–	36.9	2.3	–
Romagnolo et al.	2019	50	65.1	32	8.23 (5.13)	21.2	-	25.7 (4.4) *
Beretta et al.	2018	28	72.5	35.7	–	23.92	1.92	28.5 (1.7)
Almeida et al.	2016	229	70.7	45.8	No falls: 4.8 (3.6)	32.55	2.75	–
					Falls: 8.6 (5.4)			
Cole et al.	2016	81	68.1	36.4	6.1 (0.5)	34.7	1.9	–
Custodio et al.	2016	59	67	40.7	6	24	–	–
Gazibara et al.	2016	120	60	66.6	4	–	2.25	–
Heinzel et al.	2016	40	64.3	62.4	No falls: 3.5 (2.6)	32.6	2.45	No falls: 26.7 (2.4)
					Falls: 5.5 (3.1)			Falls: 26.9 (2.2)
Lord et al.	2016	121	68.4	61.4	0–0.2	24.1	2.8	No falls: 25.9 (3.0)
								Falls: 25.0 (3.4)
Sakushima et al.	2016	97	71.4	59.2	No falls: 6.1	16.2	2.65	No falls: 26.4 (3.0)
					Falls: 9.4			Falls: 26.4 (2.7)
Schlenstedt et al.	2016	85	67.1	33.3	No falls: 6.9 (5.2)	42.1	2.65	–
					Falls: 9.3 (6.5)			
Almeida et al.	2015	130	70.3	41.8	4.9 (3.6)	26.8	2.5	29.2 (1.2)
Duncan et al.	2015	171	67.0	44	No falls: 4.8 (3.8)	32.48	–	–
					Falls: 6.6 (4.2)			
Gazibara et al.	2015	120	61.4	–	–	–	2	–
Hoskovcová et al.	2015	45	67.2	24.4	10.2 (3.4)	22.6	2.6	24.2 (3.3) *
Kataoka and Ueno	2015	85	71.3	–	4.8	41.1	2.8	26.8 (3.2)
Hiort et al.	2014	124	70.4	49	6.9 (4.3)	20.3	–	27.3 (3.7)
Kataoka et al.	2014	26	65.3	46.2	No falls: 72.6 (69.0)†	18.65	–	No falls: 28.4 (1.5)
					Falls: 88.0 (61.2)†			Falls: 26.1 (3.3)
Mak et al.	2014	144	63.4	38.5	No falls: 7.1 (4.8)	29.9	2.55	–
					Falls: 9.5 (7.8)			
Duncan et al.	2013	80	68.2	41.3	–	41.3	2.5	–
Kim et al.	2013	119	65.5	56.5	No falls: 1.1 (0.5)	19.75	–	No falls: 26.6 (2.6)
					Falls: 1.3 (0.6)			Falls: 26.6 (2.2)
Mak et al.	2013	110	62.9	22	No falls: 6.7 (4.4)	26.2	2.75	No falls: 28.0 (2.3)
					Falls: 9.0 (6.2)			Falls: 27.8 (2.7)
Paul et al.	2013	205	67.8	41	No falls: 5.4 (4.0)	24.65	–	No falls: 29.2 (1.0)
					Falls: 8.7 (6.5)			Falls: 28.8 (1.3)
Camicioli et al.	2010	52	71.5	42.3	–	17.7	2.25	28.0 (1.75)
Allcock et al.	2009	176	71.2	37.2	7.2	19	–	25.1 (3.5)
Latt et al.	2009	113	66	41.6	–	–	–	No falls: 12.9%^a^
								Falls: 33.3%^a^
Mak et al.	2009	70	63.4	49.8	No falls: 7.2 (4.2)	25.4	2.9	–
					Falls: 7.2 (4.2)			
Wood et al.	2002	109	74.7	52.3	3 (1–31)	32.5	2	No falls: 29 (19–30)
								Falls: 27 (0–30)
Gray et al.	2000	118	–	38	–	50.5	2.55	–

*Data is provided as mean (SD) or median (Q1 - Q3). F* female, *HY* Hoehn & Yahr scale, PD, Parkinson’s disease

^†^ Disease duration is represented in months

* Cognition was tested with MoCA instead of MMSE

^a ^Proportion of PD patients with MMSE score ≤ 27/30

### Outcome definition and measurement

Falls were defined in most studies as “*an unexpected event in which the person comes to rest on the ground, floor, or lower level*” or a similar definition. However, seven studies did not provide a specific definition for falls [20, 22, 33, 35, 39, 43, 49]. In all studies, for quantifying or assessing falls in PD patients, the participants prospectively recorded falls on a diary or calendar. The researchers contacted the participants during follow-up by phone or by face to face interviews to register fall incidence. Five studies used recurrent falls as the unique outcome, two studied falls and recurrent falls, one the occurrence of first fall, and two studied falls and near falls (Table 1).

### Statistical models

Most studies used logistic regression to determine the factors associated with falling. Some of these studies extended their analyses using ROC Curves to assess model performance. Two studies opted for multivariate Poisson regression [22, 51], while one study employed negative binomial regression [30] to identify factors associated with the frequency of falls. In contrast, another study used Cox Proportional Hazard analysis [48] to assess the time from study enrollment to first fall. Furthermore, a chi-squared test [23] was used for exploratory analyses of factors that increased the risk of falls.

### Confounding factors

Twenty-six studies took confounding factors into consideration. The most frequently considered confounding factors encompassed age, sex, parameters related to walking (such as stride length or gait cycle time), prior fall history, disease duration, disease severity (measured by means of H&Y or UPDRS III scale), FoG, depression and anxiety levels, physical activity, levodopa-equivalent daily dose (LEDD, mg/day), or balance assessments (Activities-specific Balance Confidence [ABC] or Tinetti scales). The confounding factors considered in each study varied both in terms of quantity and the specific factors that were included, depending on the adopted criteria in each work. For instance, certain studies used a criterion whereby a confounding factor needed to achieve a specific level of statistical significance in univariate analyses before being incorporated into multivariable analyses.

### Factors associated with falls

**Fig. 2:**
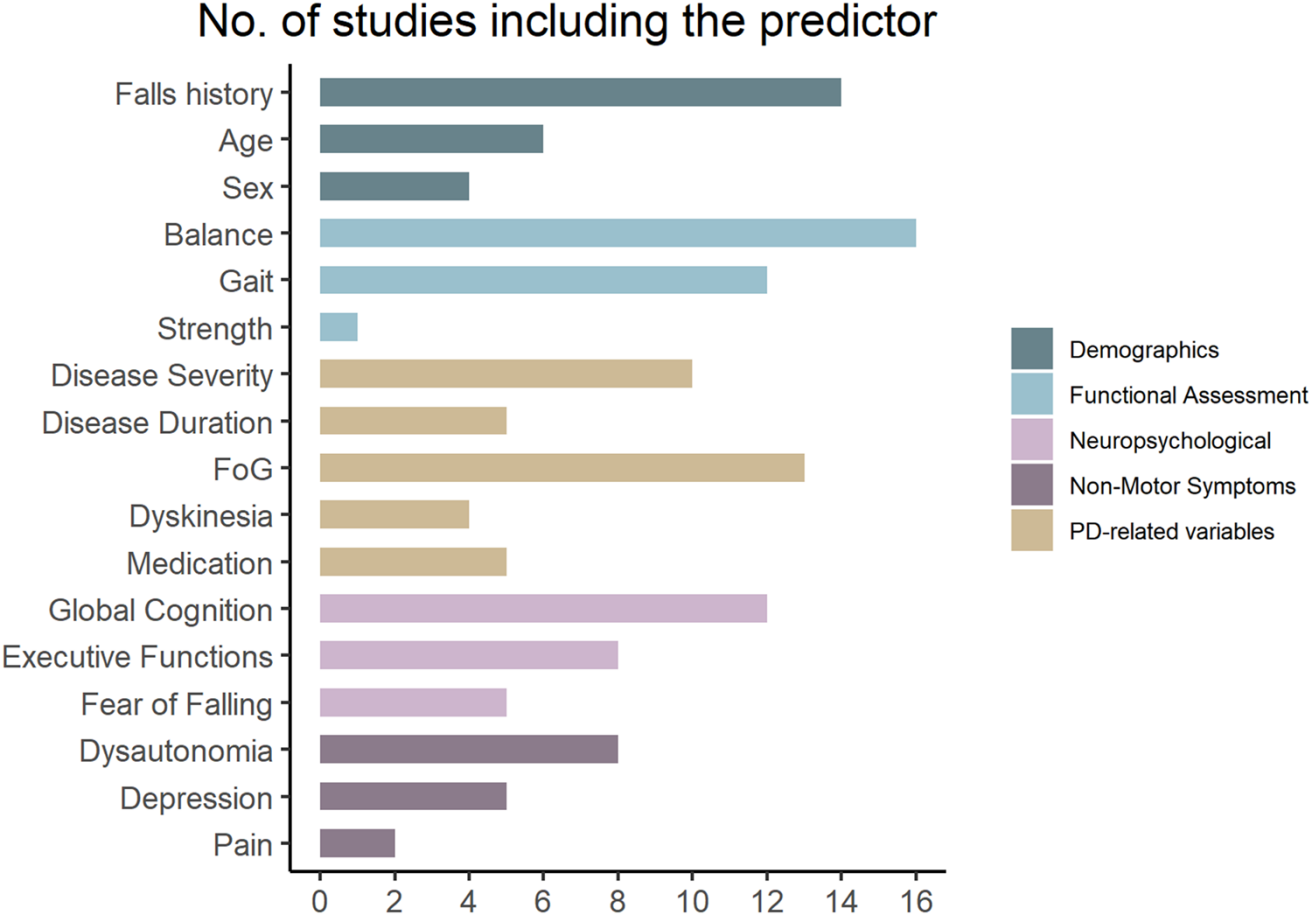
Number of prognostic factors included in the studies from this systematic review

The most extensively investigated prognostic factors for falls were history of falls, balance and gait, and FoG, followed by gait parameters, disease duration, disease severity, and global cognition (Fig. 2). Demographic factors were considered in less than 50% of included studies.

### History of falls

In relation to the history of falls, 14 studies examined this predictive variable, with the majority of them reaching the consensus that prior falls were linked to subsequent fall incidents [10, 20, 22, 25, 35, 40, 44, 50, 51, 53]. Nevertheless, it is noteworthy that the fear of falling did not exhibit an association with falls [28, 40, 41, 46, 49].

### Balance and gait parameters

The most extensively employed clinical evaluations for gait and balance include the 10-m walking test (10-MWT) [21, 36, 50, 51], which evaluates mobility at comfortable and maximal walking speed, Timed Up and Go test [10, 21, 31, 42], the Tinetti Balance Assessment evaluating both gait and balance [21, 46, 49], the 7-item Berg Balance Scale quantifying static and dynamic balance during specific movement tasks [21, 27, 31], Functional Reach Test measuring the maximal distance an individual can reach forward from a standing position [21, 25, 31], Activities-specific Balance Confidence (ABC) scale [31, 34, 40, 41, 43, 48], Mini-Balance Evaluation System Test (Mini-BESTest) [27, 29, 37, 41], Dynamic Gait Index assessing adaptability of balance during ambulation in the presence of external demands [31], and the Retropulsion test [50, 51]. Gait and balance were assessed in 21 studies.

In the included articles, regardless of the chosen assessment tool or questionnaire, a consensus emerged from 11 out of 16 studies assessing balance [21, 27, 29, 33, 34, 37, 41, 43, 46, 48, 51], and from 9 out of 12 studies assessing gait [10, 21, 22, 24, 46, 48, 50, 51, 53]. These studies showed that gait disorder and poor balance were associated with future falls. On the other hand, balance confidence measured with ABC-16 questionnaire consistently demonstrated to be significantly associated with falls [34, 40, 41, 43]. The one study that failed to find such an association was the one whose outcome was the occurrence of first fall [48].

In our systematic review, a variety of gait measurement methods have been employed, with particular focus on the instrumental evaluations conducted by Lord [48] and Hoskovcová [22]. These evaluations comprehensively assessed gait parameters and revealed a strong association between gait speed and the likelihood of future falls. Conversely, Duncan et al. [53] assessed gait using the 10-MWT, while Paul et al. [25] based their evaluation on self-selected walking pace. Both studies concluded that these gait parameters serve as reliable indicators for predicting falls. However, a study conducted by Kataoka et al.. [46] suggested that gait speed alone may not be a reliable predictor of falls. It is important to highlight that this particular study had a small sample size of only 26 participants, which could potentially constrain the ability to formulate definitive conclusions.

### Freezing of Gait

Thirteen studies explored the association between FoG and future falls. The FoGQ scale emerged as the predominant assessment tool within the reviewed articles for analyzing FoG [29, 41, 50, 53], although certain studies alternatively employed UPDRS II item 14 [22, 31, 52] or inquired about prior instances of FoG [10, 20]. However, the findings concerning its predictive efficacy for falls were contradictory. A study using Falls Efficacy Scale International Questionnaire (FES-IQ) [35], history of FoG [10, 20], FoG item from UPDRS scale [31, 52] and one study using FoG Questionnaire [25], proved FoG to be significant predictors of falls. Contrarily, half of the studies yielded opposing outcomes. As a result, the association between FoG and falls remains unclear and additional research in this area is required.

### Demographic factors

Demographic factors have not been included in many studies, but those introducing them as confounding variables have concluded that age and sex were not significantly related to falls [10, 22, 26, 30, 39, 41]. Other demographic factors, such as the socioeconomic status, have not been explored in the selected articles.

### Disease-related variables

In addition to biometric measurements, several studies have investigated the predictive nature of disease-related variables in relation to falls. These variables include dyskinesia, disease duration, motor severity, disease stage, postural asymmetry, and medication. Dyskinesias disrupt motor control and gait patterns and, therefore, have been considered as prognostic factor for falls. However, in this systematic review, the four studies including dyskinesias as prognostic factor concluded that they were not predictors of falls [22, 35, 50, 51]. However, there is conflicting information regarding the predictive role of disease duration or severity. All incorporated studies concur that the H&Y scale serves as a valuable tool for predicting falls [20, 30, 48], whereas the ability of UPDRS III in isolation to prognosticate falls is still controversial [30, 40, 41, 49]. In regard to disease duration, divergent outcomes emerge, and it is difficult to draw conclusion from the current evidence [22, 35, 39, 44, 47]. One study focusing on postural asymmetry concluded that it was a predictor of falls [33]. Out of the five studies including LEDD as a prognostic factor for falls, three studies concluded that future fallers tend to have higher initial doses of levodopa [22, 39, 52], and one study concluded that the proportion of fallers with LEDD < 500 at baseline was significantly smaller [10], whereas in the remaining study [31] the daily LEDD was near significance for predicting recurrent fallers.

### Non-motor symptoms

This review also gathered information about neuropsychological and other non-motor symptoms as prognostic factors for falls. The most prominent symptoms analyzed were cognition, depression, and dysautonomic symptoms. Executive functions were found to be predictive of falls in five out of eight studies that assessed it [28, 30, 41, 46, 49], whereas global cognition found not to be a significant prognostic factor for falls in seven out of eleven studies [20, 22, 35, 46, 48, 49, 52]. PD patients with cardiac autonomic neuropathy [42] or mild urinary urgency [26] seem to be at higher risk of falls. Contrarily, dizziness or the presence of symptoms of orthostatic hypotension were not predictors of falls [10, 23, 30, 39, 44, 48]. Regarding depression, five studies analyzing this factor as a prognostic indicator for falls found that depression was not related to falls [28, 40, 41, 48], except one study showing the opposite result [39]. Lastly, Gazibara et al.. [38, 45] reported that health-related quality of life questionnaire could also predict the occurrence of falls within 1 year of follow-up. Specifically, PD patients reporting poor physical functioning or vitality in the questionnaire were at higher risk of falls.

### Quality of included studies

**Fig. 3:**
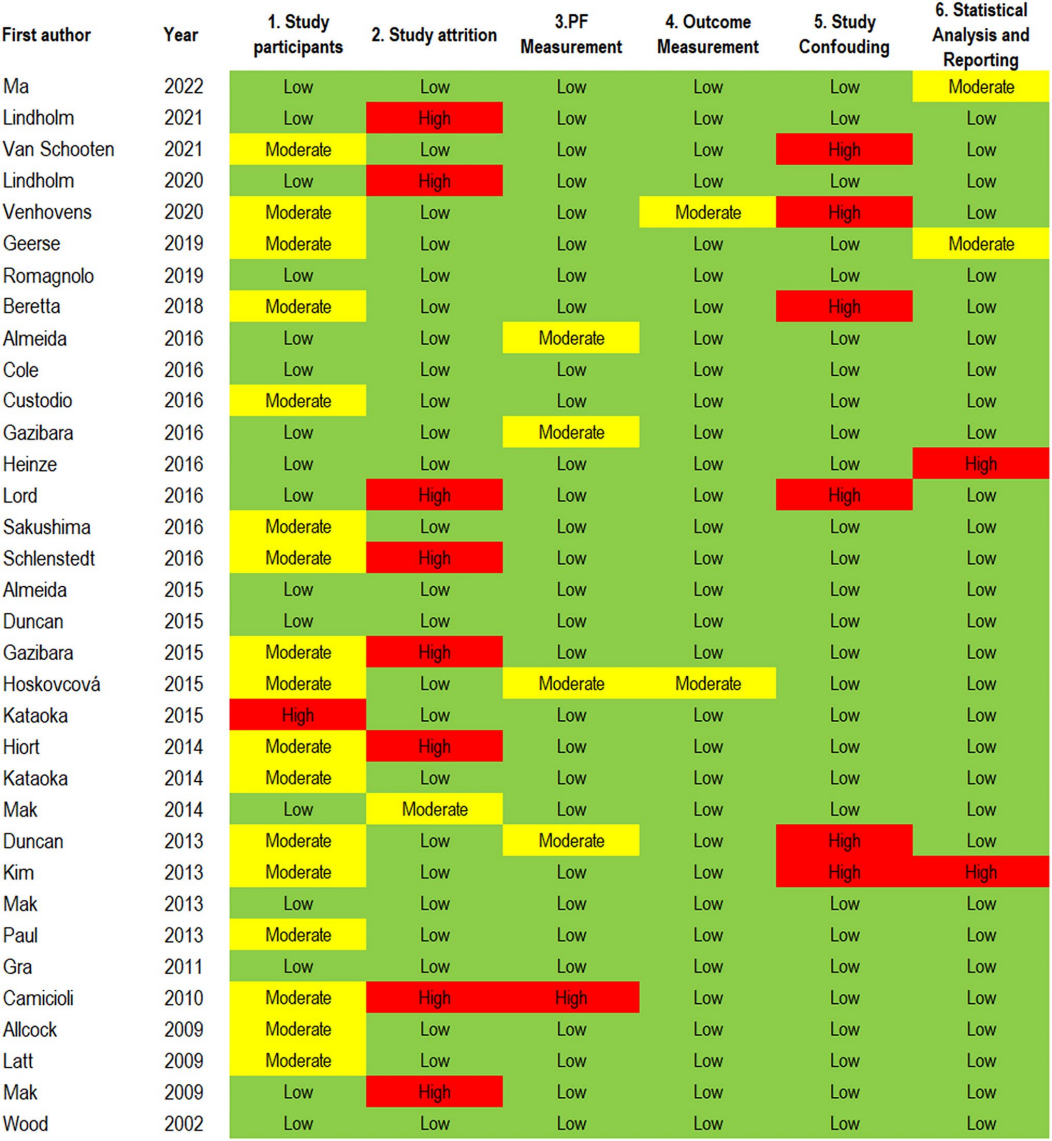
Risk of bias assessment with QUIPS tool

Using the QUIPS tool, 18 studies scored high at least in one domain from which 3 studies scored high in two domains [20, 39, 48]. The most frequently noted sources of high bias were Study Attrition (*n* = 8), and Confounding Factors (*n* = 6) categories. Confounding bias occurred when studies did not adjust for relevant, potentially confounding variables in multivariable models. Studies without an adequate strategy to address substantial missing data accounted for Study Attrition bias. Moderate risk of bias was observed in 17 studies regarding Study Participant category. Sources of bias related to study participation were generally due to insufficient clinical description of the sample (Fig. 3).

## Discussion

In this systematic review, we reviewed the current literature, specifically focusing on prospective studies that met our inclusion criteria, to identify the factors predicting falls in PD. From the interpretation of the included studies we have reached several conclusions: (1) strong evidence was found for poor balance, gait disorders, and history of falls at baseline as prognostic factors for falls in PD; (2) our review showed limited and conflicting evidence with regard to FoG, disease severity, disease duration or frontal cognitive abilities as potential predictors for falls; (3) no evidence was found for the association of baseline dyskinesias, global cognitive impairment, orthostatic symptoms, age or sex with future falls.

Previous systematic reviews have explored fall-related factors but span more than a decade and were limited by the number of articles included. Notably, Pickering et al. in 2007 [54] examined six prospective studies, and Allen et al. [55] updated the literature in 2013 with 15 prospective and 11 retrospective studies. Both systematic reviews concluded that the history of falls was the main predictor for falls in PD. They also explored the influence of disease severity, ultimately concluding that its contribution to fall prediction was minimal, which is in line with our results. However, these early reviews lacked an exploration of the full spectrum of potential predictors, which underscores the importance of revisiting this topic with a broader scope. In the current systematic review, we expand on these prior reviews by incorporating a more substantial number of prospective studies conducted in the last decade. In this work, in contrast to previous ones, we explored the role of demographics and other non-motor symptoms, including cognition, autonomic nervous system dysfunction or depression.

In the selected 34 articles, more than 17 prognostic factors were analyzed, with notable emphasis on the history of falls, FoG, balance, and gait disorders. However, not all the included articles specified the definition of falls. Notably, seven of them lack a clear definition of falls [20, 22, 33, 35, 39, 43, 49], which may limit the interpretability of the results. Moreover, some studies used “repetitive falls”, “near-falls,” or “the occurrence of first fall” as the primary outcome. Therefore, the prognostic factors identified in each study might not necessarily coincide, given the subtle variations in outcome definition. Additionally, some studies did not account for confounding factors, while those that did incorporate them displayed substantial heterogeneity in the quantity and nature of variables considered in multivariable analyses, rendering the comparability between studies challenging.

Gait and balance assessment have emerged as pivotal functional parameters frequently analyzed in PD for their predictive capacity concerning falls. In 2018, Creaby et al. [56] investigated the biomechanical parameters associated with falls in a meta-analysis, revealing walking speed, cadence, and stride length as significant indicators of future falls. This finding is in line with our results, although we were not able to analyze the data in a meta-analysis due to the heterogeneity across studies. In recent years, the integration of sensors and wearable devices has gained momentum in fall prediction research. These technologies offer a sensitive approach to measuring gait and balance. As opposed to self-reported responses, wearable devices provide objective and continuous data, allowing for a deeper understanding of a patient’s movement patterns. Recently, wearable sensors have been employed in both healthy individuals and those with PD demonstrating their usefulness to record and analyze gait, balance, and other falls-related risk factors [57, 58]. This advancement promises to enhance the accuracy of fall prediction by capturing subtle changes in gait and balance that may go unnoticed in traditional assessments. However, the multiple parameters derived from sensors introduces complexity in data analysis and more sophisticated analytical techniques might be needed, such as machine learning algorithms, to identify potential predictors for falls.

Regarding the limitations of the current work, one important aspect pertains to the method of fall recording. Falls are self-reported by patients in diaries, which introduces a potential bias and may result in less accurate data on fall incidents. Furthermore, a quantitative analysis of the extracted prognostic factors from the articles was not performed in this study, and, therefore, no statistical data are reported. Combining the current findings with a meta-analysis would provide a higher level of scientific evidence to determine whether factors such as balance or FoG are indeed predictors of falls. However, due to the heterogeneity in the included prognostic factors, confounding factors, and reported outcomes, a descriptive analysis was more suitable. This limitation might be resolved in future studies by focusing on the analysis of specific prognostic factors for falls. Lastly, it is important to note that only prospective studies were included in this review, and there may be additional prognostic factors that have been examined in retrospective longitudinal studies, yielding different results. Nonetheless, the inclusion of prospective studies is deemed valuable, as they offer greater validity in assessing the reliability and predictive value of prognostic factors. However, these studies are usually limited by short follow-up time.

In conclusion, the prognostic factors for falls in PD that were most consistently reported as significant in the literature were previous history of falls, gait disorders, and poor balance. As the prevalence of PD continues to rise globally, elucidating robust prognostic factors for falls is paramount for informing targeted interventions and optimizing patient care. As some prognostic factors have been poorly studied in the literature, such as demographics, and the heterogeneity of confounding factors is high, more research is needed to assess the predictive values of the identified factors. In future studies, using objective instruments like wearable devices for biometric assessment for falls, freezing and gait disorders could enhance the reliability of data collection. Moreover, considering the predictive nature of the intended outcome, future studies could benefit from utilizing more advanced and robust machine learning algorithms to identify predictive factors for falls in PD. This review directs attention towards key variables warranting further investigation for developing tailored fall prevention strategies in PD.

### Supplementary Information

Below is the link to the electronic supplementary material.

Supplementary file1 (DOCX 20 KB)

## Data Availability

Data used for this systematic review are available upon reasonable request.
