# Supplementary material for: Prognostic factors for falls in Parkinson’s disease: a systematic review
Source: Acta Neurol Belg. 2023 Nov 28;124(2):395–406. doi: 10.1007/s13760-023-02428-2 (PMC10965733; doi:10.1007/s13760-023-02428-2)
Supplement: Supplementary file 1 — Supplementary file1 (DOCX 20 KB) [file 13760_2023_2428_MOESM1_ESM.docx]

**Supplementary Material**

**Search strategy**

A literature search of the MEDLINE database (via Pubmed) and ISI Web of Science (WOS) was conducted from database inception to 15^th^ August 2023. The following search string was used:

("Parkinson's disease" OR "Parkinson disease") AND (fall or falls) AND prospective AND (longitudinal OR cohort OR case-control) AND (prognos* OR predict*)

We scanned reference lists of all eligible studies and reviews derived from the protocol-driven search for identifying further possible studies [1].

[1] Greenhalgh, T. and R. Peacock, *Effectiveness and efficiency of search methods in systematic reviews of complex evidence: audit of primary sources.* BMJ, 2005. **331**(7524): p. 1064-1065.

**QUIPS tool (modified) to assess risk of bias of prognostic factors**

|  |  | YES and NO are the possible answers | | | | |  |
| --- | --- | --- | --- | --- | --- | --- | --- |
|  |  | Count the number of "NO" answers in each domain and establish a risk of bias. | | | | |  |
|  |  |  |  |  | | | |
|  |  | **YES/**  **NO** | |  | | |  |
| **1. Study participants** |  |  | | **Goal: To judge the risk of selection bias (likelihood that relationship between PF and outcome is different for participants and eligible non-participants)** |  |  |  |
| Source of target population | |  | | The source population or population of interest is adequately described |  |  |  |
| Inclusion and exclusion criteria | |  | | Inclusion and exclusion criteria are adequately described (e.g., including explicit diagnostic criteria |  |  |  |
| Baseline characteristics | |  | | The baseline study sample (i.e., individuals entering the study) is adequately described for key characteristics. |  |  |  |
|  | |  | | High risk bias: 2 "NO" or more Moderate risk bias: one "NO" Low risk of bias: all answers "YES" |  |  |  |
|  |  |  |  |  | |  |  |
| **2. Study attrition** |  |  |  | **Goal: To judge the risk of attrition bias (likelihood that relationship between PF and outcome are different for completing and non-completing participants).** |  |  |  |
| Proportion of baseline sample available for analysis | |  | | Response rate (i.e., proportion of study sample completing the study and providing outcome data) is adequate |  |  |  |
| Attempts to collect information on participants who dropped | |  | | Attempts to collect information on participants who dropped out of the study are described. |  |  |  |
| Reasons and potential impact of subjects lost to follow-up | |  | | Reasons for loss to follow-up are provided. |  |  |  |
|  | |  | | High risk of bias: >20% of missing data or the other two are "NO” Moderate risk of bias: one "NO" Low risk of bias: < %20 of missing data and the remaining "YES" |  |  |  |
|  |  |  |  |  | |  |  |
| **3. Prognostic Factor Measurement** |  |  |  | **Goal: To judge the risk of measurement bias related to how PF was measured (differential measurement of PF related to the level of outcome).** |  |  |  |
| Definition of the PF | |  | | A clear definition or description of 'PF' is provided (e.g., including dose, level, duration of exposure, and clear specification of the method of measurement). |  |  |  |
| Valid and Reliable Measurement of PF | |  | | Method of PF measurement is adequately valid and reliable to limit misclassification bias |  |  |  |
| Proportion of data on PF available for analysis | |  | | If the proportion of the study sample that has missing data for PF variable is >20%, the answer is "NO" |  |  |  |
| Method used for missing data | |  | | Appropriate methods of imputation are used for missing 'PF' data. |  |  |  |
|  | |  | | High risk bias: 2 "NO" or more Moderate risk bias: one "NO”  Low risk of bias: all answers "YES" |  |  |  |
|  |  |  |  |  | |  |  |
| **4. Outcome measurement** |  |  |  | **Goal: To judge the risk of bias related to the measurement of outcome (differential measurement of outcome related to the baseline level of PF).** | |  |  |
| Definition of the outcome | |  | | A clear definition of outcome is provided, including duration of follow-up and level and extent of the outcome construct. |  |  |  |
| Valid and reliable measurement of outcome | |  | | The method of outcome measurement used is adequately valid and reliable to limit misclassification bias |  |  |  |
| Method and setting of outcome measurement | |  | | The method and setting of outcome measurement is the same for all study participants. |  |  |  |
|  | |  | | High risk bias: 2 "NO" or more Moderate risk bias: one "NO" Low risk of bias: all answers "YES" |  |  |  |
|  |  |  |  |  | |  |  |
| **5. Study confounding** |  |  |  | **Goal: To judge the risk of bias due to confounding (i.e. the effect of PF is distorted by another factor that is related to PF and outcome).** | |  |  |
| Measurement of confounders | |  | | Important confounders are measured and are the same for all subjects. |  |  |  |
| Method used for missing data | |  | | Measurement of all important confounders is adequately valid and reliable |  |  |  |
| Appropriate accounting for confounding in study design | |  | | Important potential confounders are accounted for in the study design (e.g., stratification) |  |  |  |
| Appropriate accounting for confounding in analysis | |  | | Important potential confounders are accounted for in the analysis (i.e., appropriate adjustment). |  |  |  |
|  | |  | | High risk bias: 2 "NO" or more Moderate risk bias: one "NO" Low risk of bias: all answers "YES" |  |  |  |
|  |  |  |  |  | |  |  |
| **6. Statistical Analysis and Reporting** |  |  |  | **Goal: To judge the risk of bias related to the statistical analysis and presentation of results.** | |  |  |
| Presentation of analytical strategy | |  | | There is sufficient presentation of data to assess the adequacy of the analysis |  |  |  |
| Model development strategy | |  | | The selected statistical model is adequate for the design of the study. |  |  |  |
| Reporting of results | |  | | There is no selective reporting of results |  |  |  |
|  | |  | | High risk bias: 2 "NO" or more Moderate risk bias: one "NO" Low risk of bias: all answers "YES" |  |  |  |
